# Onchocerciasis-associated epilepsy in the Democratic Republic of Congo: Clinical description and relationship with microfilarial density

**DOI:** 10.1371/journal.pntd.0007300

**Published:** 2019-07-17

**Authors:** Joseph Nelson Siewe Fodjo, Michel Mandro, Deby Mukendi, Floribert Tepage, Sonia Menon, Swabra Nakato, Françoise Nyisi, Germain Abhafule, Deogratias Wonya’rossi, Aimé Anyolito, Richard Lokonda, An Hotterbeekx, Robert Colebunders

**Affiliations:** 1 Global Health Institute, University of Antwerp, Antwerp, Belgium; 2 Ministry of Health, Ituri, Democratic Republic of Congo; 3 Mont Amba Neuropsychopathologic Center, University of Kinshasa, Kinshasa, Democratic Republic of Congo; 4 Ministry of Health, Bas-Uélé, Democratic Republic of Congo; 5 Centre de Recherche en Maladies Tropicales de l'Ituri, Rethy, Democratic Republic of Congo; 6 National Onchocerciasis Control Program, Ituri, Democratic Republic of Congo; 7 Hôpital Général de Référence de Logo, Ituri, Democratic Republic of Congo; University of Buea, CAMEROON

## Abstract

**Background:**

High epilepsy prevalence and incidence were observed in onchocerciasis-endemic villages in the Democratic Republic of Congo (DRC). We investigated the clinical characteristics of onchocerciasis-associated epilepsy (OAE), and the relationship between seizure severity and microfilarial density.

**Methods:**

In October 2017, ivermectin-naive persons with epilepsy (PWE) were recruited from onchocerciasis-endemic areas in the Logo health zone in the DRC. Additional PWE were enrolled in the Aketi health zone, where ivermectin had been distributed annually for 14 years. Past medical history, clinical characteristics and skin snips for *Onchocerca volvulus* detection were obtained from participants. Bivariate and multivariable analyses were used to investigate associations with microfilarial density.

**Results:**

Of the 420 PWE in the Logo health zone, 392 were skin snipped (36.5% positive). Generalized motor seizures were most frequent (392 PWE, 93.3%), and nodding seizures were reported in 32 (7.6%) participants. Twelve PWE (3.1%) presented Nakalanga features. Sixty-three (44.1%) skin snip-positive PWE had a family history of epilepsy, compared to only 82 (32.9%) skin snip-negative PWE (p = 0.027). Eighty-one onchocerciasis-infected PWE were recruited in the Aketi health zone. Positive correlations between seizure frequency and microfilarial density were observed in Logo (Spearman-rho = 0.175; p<0.001) and Aketi (Spearman-rho = 0.249; p = 0.029). In the multivariable model adjusted for age, gender, and previous treatment, high seizure frequency was associated with increasing microfilarial density in Aketi (p = 0.025) but not in Logo (p = 0.148).

**Conclusion:**

In onchocerciasis-endemic regions in the DRC, a wide spectrum of seizures was observed. The occurrence of Nodding seizures and Nakalanga features, as well as an association between seizure severity and *O*. *volvulus* microfilarial density suggest a high OAE prevalence in the study villages.

**Trial registration:**

ClinicalTrials.gov NCT03052998.

## Author summary

Several epidemiological surveys suggest that onchocerciasis (a disease resulting from an infection with the parasite *Onchocerca volvulus*) is a cause of epilepsy. We conducted a study to describe the clinical characteristics of persons with epilepsy (PWE) living in onchocerciasis-endemic villages in the Democratic Republic of Congo. In some study sites, the frequency of seizures increased with increasing number of *O*. *volvulus* microfilariae detected in the skin snips of participants. A wide spectrum of seizures was observed, including generalized tonic-clonic seizures, absence seizures, and focal seizures. Growth retardation and household clustering of PWE were common. Specific clinical presentations such as nodding seizures and Nakalanga features were encountered. These results suggest a high prevalence of onchocerciasis-associated epilepsy (OAE) in the study villages.

## Introduction

As early as the 1930s, onchocerciasis was already suspected to cause seizures [1]. A meta-analysis has reported a 0.4% increase in epilepsy prevalence, for every 10% increase in onchocerciasis prevalence [2]. Today, there is increasing evidence that onchocerciasis is a risk factor for epilepsy [3–6] and that proper onchocerciasis elimination strategies can reduce the incidence of onchocerciasis-associated epilepsy (OAE) [7]. However, the physiopathology explaining how *Onchocerca volvulus* (the parasite responsible for the clinical manifestations of onchocerciasis) may cause seizures remains unclear.

Recent studies in the Democratic Republic of Congo (DRC) have revealed a high epilepsy prevalence in hyper-endemic onchocerciasis foci, particularly where control measures are sub-optimal and transmission is ongoing [8–11]. Although specific phenotypic features of OAE such as nodding seizures (repeated, involuntary forward bobbing of the head with reduced consciousness) and Nakalanga syndrome (growth retardation, dysmorphic features and cognitive decline) have already been reported in the DRC [7,9], the full clinical spectrum of OAE in the DRC remains unknown. In a bid to further elucidate the association between epilepsy and onchocerciasis, a randomized clinical trial evaluating the effect of ivermectin on the frequency of seizures in persons with epilepsy (PWE) living in the Logo health zone was initiated in October 2017 [12] (Trial Registration Number NCT03052998; available at: www.clinicaltrials.gov). During the recruitment phase of this trial, all consenting PWE were examined and skin snipped to assess eligibility criteria. This paper describes the clinical features observed in ivermectin-naïve PWE encountered during the trial. Additional data to investigate the relationship between seizures and infection with *O*. *volvulus* were obtained from the Aketi health zone, another hyper-endemic onchocerciasis focus in the DRC with high epilepsy prevalence [10].

## Methods

### Study design

We carried out a cross-sectional, descriptive study of PWE in the Democratic Republic of Congo.

### Study sites

**Fig 1:**
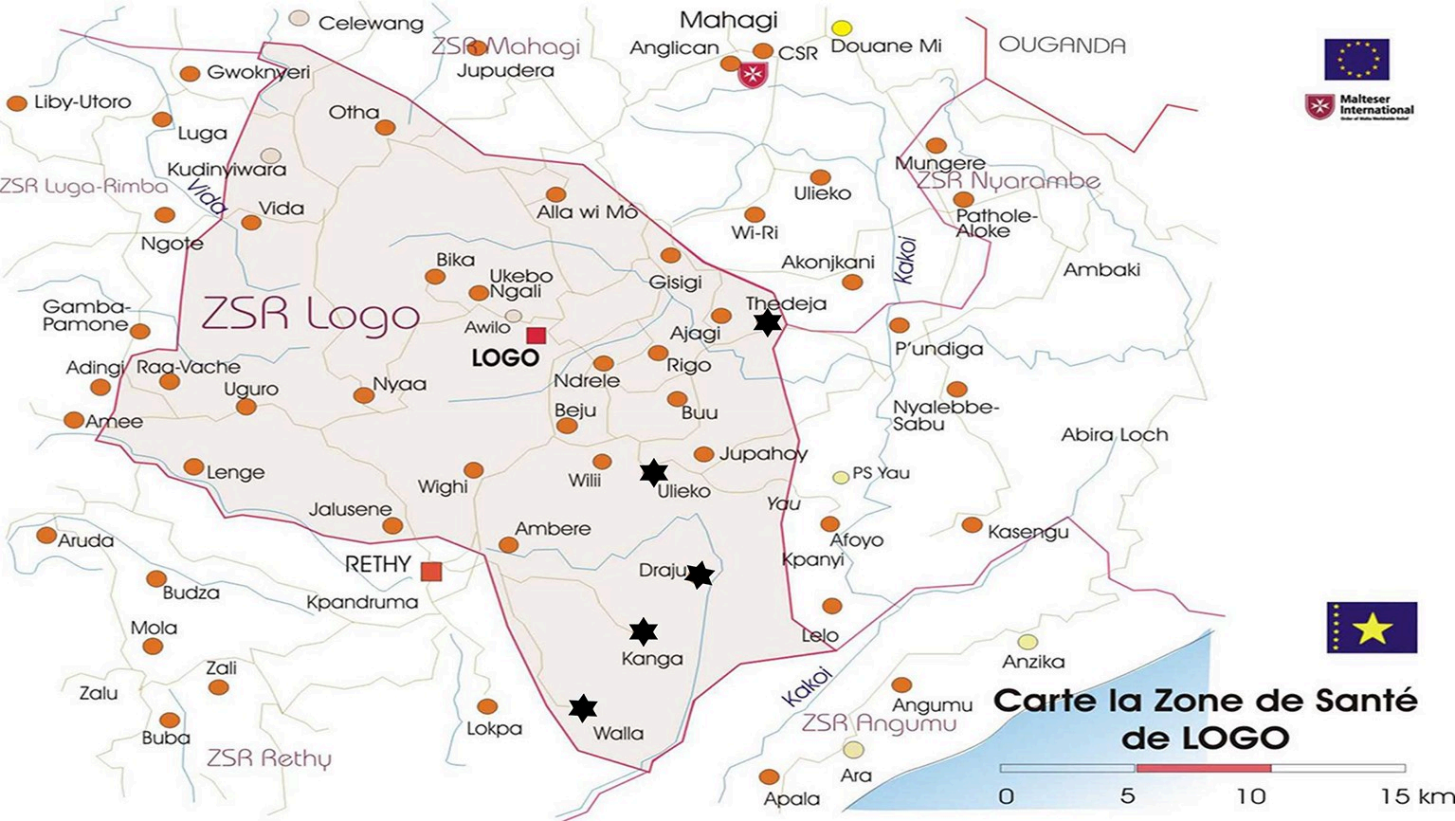
Map of the Logo health zone showing the study villages *(Source*: *Malteser International)*.

The study was conducted in two health zones in the DRC, namely Logo (in the Ituri province) and Aketi (in the Bas-Uélé province). In the Logo health zone, five onchocerciasis-endemic health areas where community-directed treatment with ivermectin (CDTI) had never been implemented were selected: Draju, Kanga, Tedheja, Ulyeko and Wala (Fig 1). In the Aketi health zone, the study sites had already benefited from 14 years of CDTI and included Wela, Makoko, and Aketi rural town. The ecology and setting was similar in all study sites; these were essentially rural communities, with several fast-flowing rivers providing suitable breeding grounds for the blackflies (*Simulium spp*), vectors of *O*. *volvulus*. The main economic activity of the residents was farming.

### Study procedures

#### In the Logo health zone

This study was conducted within the scheme of a wide program launched in October 2017, aiming to treat all the PWE in the health zone, including a clinical trial investigating the effect of ivermectin on seizures [12]. Prior to the start of the study, local authorities were contacted and the study was explained to them in detail. After obtaining their collaboration, we proceeded to recruit participants using a community-based approach. The residents of the target villages were sensitized, and persons known to have epilepsy were invited to spontaneously report to the mobile clinics set up by the research team at the health centres. Additional potential participants were referred to the clinic by community health workers who had been trained by the research team to screen persons suspected to have epilepsy in their respective villages.

All persons suspected to have epilepsy who reported to the mobile clinics were briefed on the study objectives and procedures in the local language (*Alur)*, and informed consent was provided by the participant and/or the caretaker. Upon confirmation of the epilepsy diagnosis, PWE were further interviewed and examined by a neurologist (DM) or a medical doctor trained in epilepsy (JNSF, MM, AA, RC). Participants’ weight was measured using a weighing scale, and their heights obtained with a stadiometer. Information was collected on seizure semiology, seizure frequency, past medical history, antiepileptic treatment history and family history of epilepsy. Cognitive and behavioural symptoms were grossly assessed by investigating if the participant was coherent in speech, obedient to orders or displayed any unexplained aggressive attitudes and/or wandering episodes.

Two approaches were used to assess growth retardation among our participants. For PWE below 20 years, the World Health Organization (WHO) height-for-age Z-scores were used, and any participant whose height was found below -2Z was considered to be stunted [13]. For PWE aged 20 years and above, the mean height of an adult residing in the DRC was retrieved from literature as being 157.4±7.56 cm (only women’s height was available) [14]. We therefore adopted 157.4–7.6 = 149.8 cm, as the cut-off height under which adult participants were considered to be growth retarded.

Onchocerciasis was diagnosed in two ways. Participants were initially tested for Ov16 antibodies using rapid diagnostic tests (Ov16 RDT, Standard diagnostics, Inc., Yongin-si, Gyeonggi-do, Korea). Thereafter, two skin snip samples were collected from each participant for the microscopic detection of *O*. *volvulus* microfilariae (MF). All relevant clinical and laboratory information was collected on paper and later entered in computers using the REDCap platform (https://www.project-redcap.org/), a secure web-based electronic database. The collected data was extracted and analyzed.

#### In the Aketi health zone

In January 2018, our research team recruited PWE in Wela, Makoko and Aketi rural town just before the yearly distribution of ivermectin. Community health workers and local health personnel referred suspected cases of epilepsy to a physician (FT) for confirmation. Skin snips were collected from confirmed PWE and examined for MF. The sociodemographic information, history of previous ivermectin and anti-epileptic drug use as well as seizure frequencies were obtained from participants with positive skin snips. A detailed clinical examination was not done for PWE in Aketi, because the main research objective in this health zone was to evaluate seizure frequency and MF density among PWE prior to ivermectin treatment, and to determine their response to the treatment. All collected data was entered in Microsoft Excel 2016 spreadsheets.

#### Epilepsy diagnosis and seizure classification

PWE were diagnosed in a two-step approach. Firstly, suspected cases were identified by administering a 5-item validated questionnaire [15]. Any individual who answered affirmatively to at least one question was further clerked and examined by a neurologist or a physician with training in epilepsy. Epilepsy diagnosis was confirmed according to the 2014 International League Against Epilepsy (ILAE) operational definition: two or more unprovoked seizures with at least 24 hours separating the two events [16]. All reported seizures were classified following the ILAE 2017 nomenclature [17], and the evaluation of the seizure frequency included all diagnosed seizure types. The number of seizures per month was approximated to the nearest integer. In conformity with previously proposed OAE criteria [7], any PWE who reported a sudden onset of seizures between the ages of 3–18 years without any prior psychomotor abnormality and no obvious cause of the epilepsy, was considered as having OAE.

#### Detection of *Onchocerca volvulus* microfilariae

Skin snips were taken from the left and right iliac crests of participants using a sterile Holtz corneo-scleral punch (2mm) to investigate infection with *O*. *volvulus*. The collected skin snips were incubated for 24 hours in isotonic saline in a flat-bottomed microtiter plate. The MF that emerged were counted using an inverted microscope, and the average count for both skin snips from each participant was calculated. MF densities were expressed as MF/skin snip. The same experienced laboratory technician examined the skin snips from all study sites.

#### Data analysis

Data was analysed in R version 3.5.1. Continuous variables were either expressed as mean or median/ interquartile range (IQR), and compared across groups (*O*. *volvulus-*infected vs uninfected) using the Wilcoxon rank sum test. Categorical data were expressed as proportions and compared using Chi-squared tests. The Spearman rho was used to test for correlations.

For multivariable analyses, we used seizure frequency as a proxy outcome variable for epilepsy severity. A negative binomial regression was appropriate because of the over-dispersion of the monthly seizure frequencies of participants; the superiority of this model over the ordinary Poisson regression model was confirmed by the Vuong test. The main independent variable used was MF density, with adjustments made for age, sex, and previous treatment. Any p-value less than 0.05 was considered to be statistically significant.

#### Ethical considerations

Ethical approval for the study was obtained from the ethical committee of the School of Public Health of the University of Kinshasa in the DRC (Approval number: ESP/CE/013/2018) and the ethical committee of the University of Antwerp (Registration number: B300201733350). All PWE willingly participated in the study and provided signed/thumb-printed informed consents. The identity and information of participants was kept confidential. In collaboration with the non-governmental organizations Malteser international and VZW Aketi, decentralized community-based programs were implemented to provide anti-epileptic drugs to PWE in the study sites.

## Results

### PWE in the Logo health zone

A total of 420 PWE in the Logo health zone were enrolled in the study (age range: 1–72 years). Skin snip data was available for 392 (93.3%) participants; of these, 143 (36.5%) had detectable MF (Table 1). The mean MF density was 23.2 MF/skin snip, with median: 0 (IQR: 0–9.6 MF/skin snip).

**Table 1:** Sociodemographic characteristics of PWE in the Logo health zone.

	All PWE N = 420^a^	Skin snip negative n = 249	Skin snip positive n = 143	P-value
Median age in years (IQR)	19.0 (14.0–29.0)	18.0 (13.0–29.0)	23.0 (18.0–31.0)	***< 0*.*001***
**Gender**				*0*.*776*
Number of males: n (%)	218 (51.9)	129 (51.8)	72 (50.3)	
Number of females: n (%)	202 (48.1)	120 (48.2)	71 (49.7)	
Level of education*				*0*.*263*
None: n (%)	155 (37.5)	85 (35.0)	49 (34.5)	
Primary: n (%)	218 (52.8)	129 (53.1)	84 (59.2)	
Secondary: n (%)	39 (9.4)	28 (11.5)	9 (6.3)	
University: n (%)	1 (0.2)	1 (0.4)	0 (0)	

^a^Includes 28 participants without skin snip results

*7 missing values

IQR: Interquartile range

**Fig 2:**
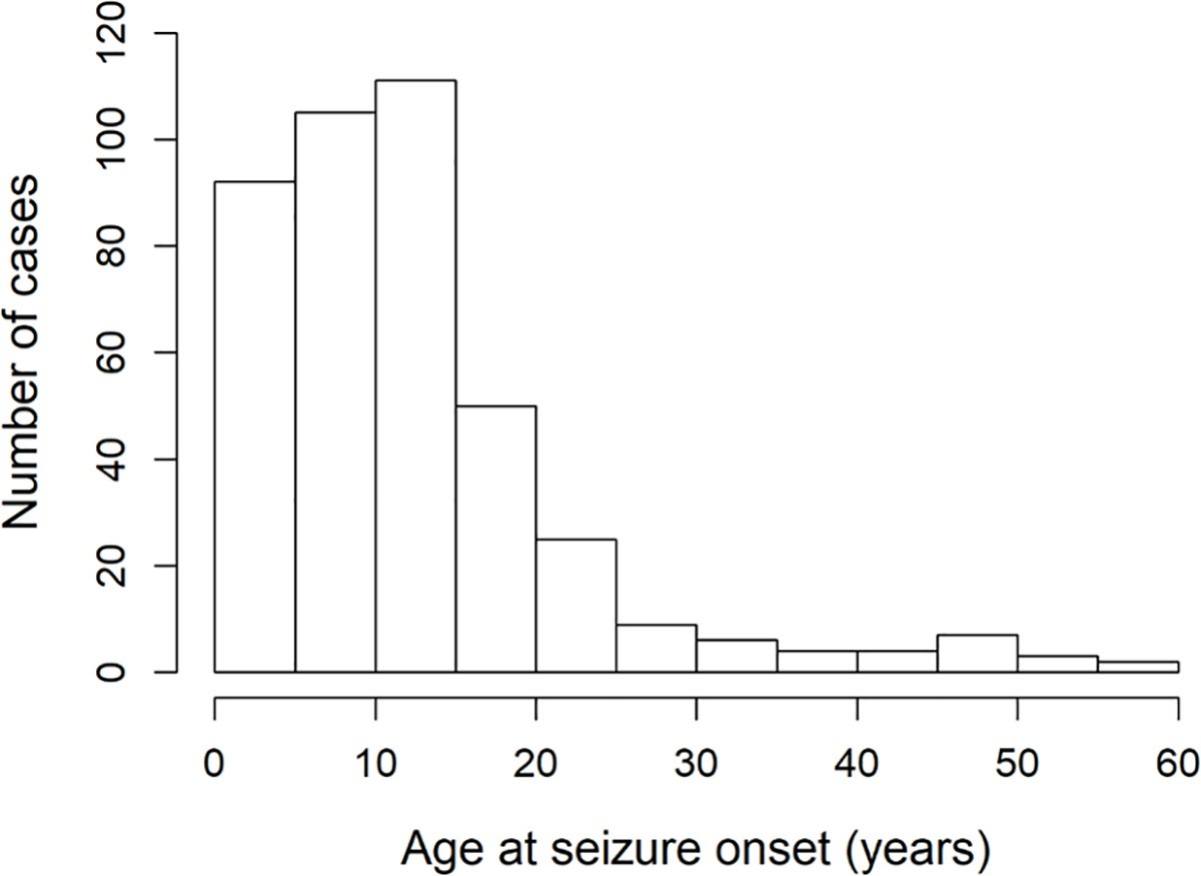
Ages of participants at seizure onset.

Epilepsy duration ranged from 0–53 years, with a median of 7 years (IQR: 3–14). In 51 (12.3%) participants, the duration of epilepsy was ≤1 year (new cases of epilepsy). The median age for epilepsy onset was 11 years, with 308 (73.3%) PWE experiencing the first epileptic seizure between 3–18 years (Fig 2).

Generalized motor seizures were reported in 392 (93.3%) PWE, and included 388 (92.1%) with generalized tonic-clonic seizures, 2 (0.5%) generalized myoclonic seizures, 2 (0.5%) generalized atonic seizures (“drop attacks”), and 1 (0.2%) generalized tonic seizures. Nodding seizures were reported in 32 (7.6%) participants. One hundred and sixty-five (39.3%) PWE experienced more than one seizure type. Table 2 summarizes the clinical presentations of participants in the Logo health zone, stratified by skin snip status; the denominators may vary for the different parameters because of missing data.

**Table 2:** Clinical characteristics of PWE in the Logo health zone.

	All PWE^a^ N = 420	Skin snip negative n = 249	Skin snip positive n = 143	P-value
**Anthropometric characteristics**				
Growth retardation: n (%)	122/386 (31.6)	71/216 (32.9)	41/142 (28.9)	*0*.*425*
**Seizure characteristics**				
Seizure frequency per month (IQR)	2.0 (1.0–3.0)	2.0 (0–3.0)	2.0 (1.0–4.0)	***0*.*001***
Age at seizure onset in years (IQR)*	11.0 (6.3–6.0)	10.0 (6.0–15.2)	13.0 (9.0–17.0)	***0*.*001***
Epilepsy duration in years (IQR)*	7.0 (3.0–14.0)	7.0 (4.0–12.6)	10.0 (3.0–16.8)	*0*.*052*
Generalized motor seizures: n (%)	392/420 (93.3)	227/248 (91.5)	138/143 (96.5)	*0*.*057*
Absence seizures: n (%)	168/420 (40.0)	101/248 (40.7)	62/143 (43.4)	*0*.*603*
Nodding seizures: n (%)	32/420 (7.6)	16/248 (6.5)	14/142 (9.9)	*0*.*223*
Focal motor seizures, conserved awareness: n (%)	8/386 (2.1)	3/216 (1.4)	5/142 (3.5)	*0*.*189*
Focal motor seizures, reduced awareness: n (%)	34/386 (8.8)	17/216 (7.9)	16/142 (11.3)	*0*.*278*
Focal to bilateral tonic-clonic seizures: n (%)	22/359 (6.1)	13/217 (6.0)	9/142 (6.3)	*0*.*908*
Focal non-motor seizures, mainly visual hallucinations: n (%)	74/349 (21.2)	47/224 (21.0)	27/125 (21.6)	*0*.*896*
Unclassified seizures: n (%)	1/358 (0.3)	1/216 (0.5)	0/142 (0)	*NA*
**Clinical and laboratory findings**				
Itching: n (%)	141/414 (34.1)	83/245 (33.9)	57/142 (40.1)	*0*.*222*
Palpable nodules: n (%)	24/406 (5.9)	8/236 (3.4)	14/143 (9.8)	***0*.*010***
Burn scars: n (%)	98/417 (23.5)	60/249 (24.1)	38/142 (26.8)	*0*.*554*
Cognitive impairment: n (%)	143/415 (34.5)	87/245 (35.5)	48/143 (33.6)	*0*.*705*
Abnormal behaviour: n (%)	47/120 (39.2)	27/69 (39.1)	18/47 (38.3)	*0*.*931*
Spinal/thoracic deformity: n (%)	5/385 (13.0)	2/216 (0.9)	3/142 (2.1)	*0*.*341*
Nakalanga features**: n (%)	12/386 (3.1)	7/216 (3.2)	5/142 (3.5)	*0*.*877*
Positive Ov16 rapid test result: n (%)	127/362 (35.1)	49/211 (23.2)	76/123 (61.8)	***< 0*.*001***
OAE criteria met [7]: n (%)	284/420 (67.6)	165/249 (66.3)	110/143 (76.9)	***0*.*027***

^a^Includes 28 participants without skin snip results

*2 missing data

**Growth retardation, delayed sexual development, cognitive impairment, and/or deformities [18]

IQR: Interquartile range; OAE: Onchocerciasis-associated epilepsy; NA: Not available

Among the 284 PWE (67.6%) who met the OAE diagnostic criteria, 110/275 (40.0%) and 99/150 (39.8%) were positive for skin snips and Ov16 rapid tests, respectively. Only 258 of these OAE participants had complete data for both Ov16 and skin snip results, and 147 (57.0%) of them were positive for at least one onchocerciasis test. The monthly seizure frequency among PWE who met the OAE criteria (2.0, IQR: 1.0–4.0) was higher than for non-OAE PWE (1.5, IQR: 1.0–2.0); p = 0.007. Moreover, a higher mean MF density was observed among the PWE who fulfilled the OAE criteria (25.3 MF/skin snip) compared to other participants (18.4 MF/skin snip); p = 0.021.

Nodding seizures were reported in 32 (7.6%) PWE. When compared with PWE without a history of nodding seizures, PWE with nodding seizures were younger (median ages: 16.0 years (IQR: 13.0–19.0) vs 20.0 years (IQR: 14.2–29.0); p = 0.01), had a higher seizure frequency (3.0 seizures/month (IQR: 2.0–16.2) vs 2.0 seizures/month (IQR: 1.0–3.0); p<0.001), were more often cognitively impaired (71.9% vs 31.2%; p<0.001), and had a higher prevalence of delayed secondary sexual development (11.1% vs 2.5%; p = 0.01). Age at seizure onset was not significantly different among participants who reported nodding seizures (age at onset: 9.5 years; IQR: 6.0–12.0) compared to those who did not (11.0 years; IQR: 7.0–17.0); p = 0.09.

Twelve PWE presented with Nakalanga features (Table 3); in all those for whom the age at epilepsy onset was known, the first seizures appeared between 3 and 12 years. Two thirds (8/12) of PWE with Nakalanga features were positive for at least one onchocerciasis test.

**Table 3:** Clinical features and onchocerciasis diagnosis in PWE with the Nakalanga features.

Case	Socio-demography		Anthropometry			Seizure history				Other clinical manifestations			OAE	Onchocerciasis diagnosis		
	Sex	Age	Height (cm)	Height-for-age Z-score^1^	Summary	Age at onset	Seizure types	Frequency (monthly)	Epileptic siblings	Cognitive impairment	Sexual development	Deformity	Criteria met^2^	Number of nodules	MF density^3^	Ov16
1	Female	16 years	145	-2.6	Moderate stunting	4 years	Generalized tonic clonic; Absence; focal sensory	12	0	No	Mature breast No pubic hair	None	Yes	0	0	+
2	Male	22 years	140	ND	Below the mean adult height*	8 years	Generalized tonic clonic	90	2	Yes	No pubic hair	Lordosis; facial dysmorphia	Yes	0	155.5	+
3	Female	18 years	143	-3.0	Severe stunting	NA	Generalized tonic clonic; Absence; focal sensory	3	1	Yes	Mature breast No pubic hair	None	NA	2	159.5	-
4	Male	18 years	144	-4.3	Severe stunting	12 years	Nodding; absence	3	0	Yes	No pubic hair	None	Yes	0	0	+
5	Female	30 years	136	ND	Below the mean adult height*	5 years	Generalized tonic clonic; Absence; Nodding; focal sensory	0	0	Yes	Mature breast Pubic hair present	None	Yes	0	0	-
6	Male	29 years	137	ND	Below the mean adult height*	12 years	Generalized tonic clonic	3	0	Yes	No pubic hair	Kyphosis, facial dysmorphia	Yes	0	0	-
7	Female	19 years	136	-4.2	Severe stunting	5 years	Generalized tonic clonic; Absence	3	2	No	Immature breast No pubic hair	None	Yes	0	27.5	NA
8	Female	19 years	152	-1.7	Low height, not stunted	7 years	Generalized tonic clonic; Absence	5	0	Yes	Mature breast Pubic hair present	None	Yes	0	0	-
9	Female	19 years	142	-3.2	Severe stunting	8 years	Generalized tonic clonic; Absence; Nodding; focal seizure + impaired awareness	15	0	Yes	Mature breast No pubic hair	Kyphosis	Yes	0	0.5	-
10	Male	24 years	155	ND	Below the mean adult height*	8 years	Generalized tonic clonic; Absence; focal sensory	15	0	Yes	No pubic hair	Thoracic deformity	Yes	1	126.5	NA
11	Female	19 years	150	-2.1	Moderate stunting	3 years	Generalized tonic clonic; Absence	16	2	Yes	Mature breast Pubic hair present	None	Yes	0	0	+
12	Female	27 years	145	ND	Below the mean adult height*	5 years	Generalized tonic clonic	2	0	Yes	Mature breast Pubic hair not examined	None	Yes	0	0	-

^1^For participants younger than 20 years, based on the World Health Organization growth curves [13]

^2^Based on previously published criteria [7]

^3^Number of microfilariae per skin snip

*Mean height of a female adult in the Democratic Republic of Congo: 157.4 cm [14]

MF: microfilaria; NA: Not available; ND: Not done; OAE: Onchocerciasis-associated epilepsy

Table 4 summarizes the past history of PWE in the Logo health zone. Overall, 136 probable neurological events were reported prior to epilepsy onset, of which 62 (45.6% of the events) were seizures with fever. Of the 288 PWE who reported ever taking anti-epileptic drugs (AED), the molecules used included: phenytoin (91 PWE, 31.6%), phenobarbital (13 PWE, 4.5%) and carbamazepine (1 PWE, 0.3%). The remaining participants could not recall the name of the AED used. Participants with a family history of epilepsy had more positive skin snips (44.1% vs 32.9%; p = 0.027) and higher mean MF densities (31.7 MF/skin snip vs 18.2 MF/skin snip; p = 0.007) when compared with PWE without a relevant family history.

**Table 4:** Past history of PWE in the Logo health zone.

	All PWE^a^ n (%)	Skin snip negative n (%)	Skin snip positive n (%)	P-value
Head trauma with loss of consciousness	6/413 (1.5)	6/246 (2.4)	0/139 (0)	*NA*
Probable perinatal asphyxia*	20/380 (5.3)	11/233 (4.7)	5/132 (3.8)	*0*.*687*
Meningitis/encephalitis	4/412 (1.0)	4/246 (1.6)	0/138 (0)	*NA*
Malaria	38/384 (9.9)	27/245 (11.0)	11/139 (7.9)	*0*.*389*
Measles	6/350 (1.7)	5/212 (2.4)	1/138 (0.7)	*0*.*234*
Seizure with fever in childhood	62/380 (16.3)	38/234 (16.2)	16/120 (13.3)	*0*.*473*
Ever used anti-epileptic drugs	288/418 (68.9)	171/248 (69.0)	94/143 (65.7)	*0*.*502*
Ever used traditional medicine	167/385 (43.4)	99/215 (46.0)	59/142 (41.5)	*0*.*403*
Family history of epilepsy**	151/420 (36.0)	82/249 (32.9)	63/143 (44.1)	***0*.*027***

^a^Includes 28 participants without skin snip results

*Difficult labour and/or birth by emergency caesarean section

**Epilepsy in a first degree relative, either parent or sibling

NA: Not available

**Fig 3:**
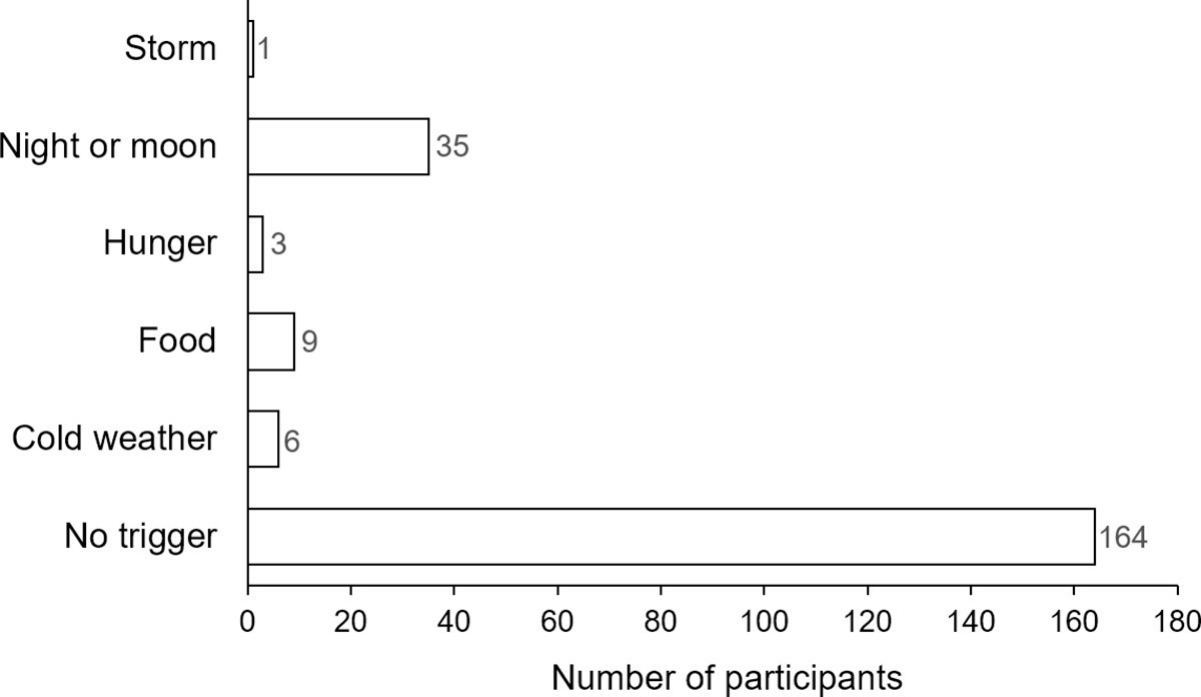
Seizure triggers among PWE in the Logo health zone.

**Fig 4:**
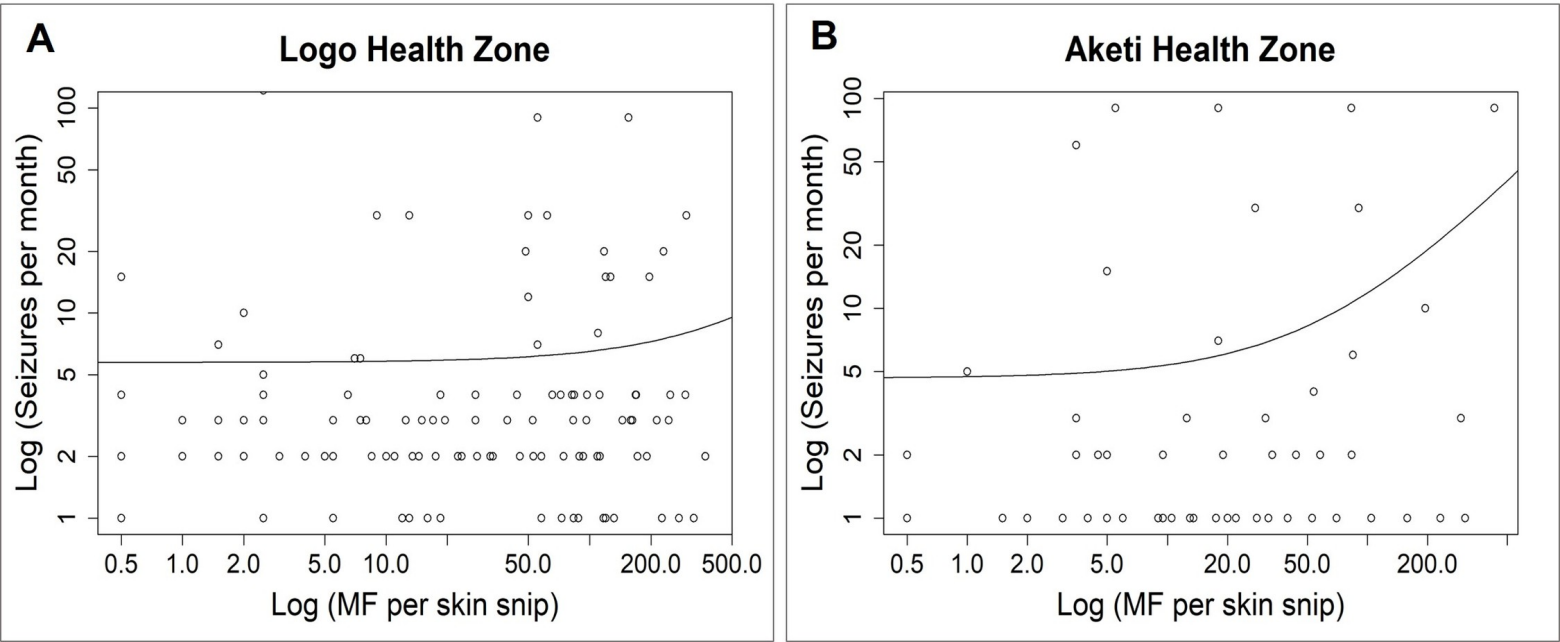
Correlation between frequency of seizures and microfilarial density among PWE. 4A shows data from PWE in Logo, while 4B shows data from PWE in Aketi. Both axes showing log values.

Different seizure triggers were identified, including food, cold weather, and storms (Fig 3). Eight of the nine PWE (88.9%) who reported food as a trigger were experiencing nodding seizures. Correlation analysis showed a positive relationship between seizure frequency and MF density among PWE in the Logo health zone: Spearman rho: 0.175; p<0.001 (Fig 4A). The multivariable analysis did not show an association between MF density and seizure frequency (Table 5).

**Table 5:** Multivariable analysis for factors associated with seizure frequency in the study sites.

	Logo Health Zone		Aketi Health Zone	
	Adj. IRR (95% CI)	*P-value*	Adj. IRR (95% CI)	*P-value*
MF density	1.002 (0.999–1.005)	*0*.*148*	1.006 (1.001–1.012)	***0*.*025***
Age	0.985 (0.974–0.996)	***0*.*013***	0.970 (0.893–1.057)	*0*.*424*
Female gender	0.964 (0.682–1.361)	*0*.*820*	2.616 (1.126–6.290)	***0*.*020***
Previous AED treatment	0.574 (0.399–0.816)	***0*.*001***	2.040 (0.620–6.126)	*0*.*121*
Previous ivermectin use	NA	*NA*	0.582 (0.240–1.346)	*0*.*189*

MF: Microfilariae

AED: Anti-epileptic drug

Adj. IRR: Adjusted incidence risk ratio

CI: Confidence interval

NA: Not applicable

### PWE in the Aketi health zone

Eighty-one onchocerciasis infected PWE (50.6% males) were recruited in the Aketi health zone; median age: 17 years (IQR: 15–20). There was one PWE (1.2%) who experienced nodding seizures in Aketi. The mean MF density was 47.0 MF/skin snip with median 10.5 (IQR: 3.5–53.0), significantly lower than the MF density of skin snip-positive PWE in Logo (p = 0.014). PWE in Aketi had fewer seizures (1.0 per month, IQR: 1.0–2.0) compared to onchocerciasis-infected PWE in Logo (p<0.001). CDTI coverage among the participants in the year prior to the study was 50/81 (61.7%), and 55 PWE (67.9%) reported previous AED use. Correlation analysis showed a positive relationship between seizure frequency and MF density (Spearman rho: 0.249, p = 0.029; Fig 4B). After adjusting for age, sex, previous AED and ivermectin use, the seizure frequency of participants was still significantly associated with MF density; p = 0.025 (Table 5).

## Discussion

To the best of our knowledge, this is the first paper describing the clinical characteristics of epilepsy and its relationship with MF density in onchocerciasis-endemic areas in the DRC. A wide spectrum of seizures was observed, with more than one third of participants reporting at least two seizure types. Nodding seizures and Nakalanga features were reported, suggesting a high prevalence of OAE in these communities as previously observed in Ituri (DRC) [9], in the Mbam valley (Cameroon) [19], Mahenge (Tanzania) [20], and Maridi (South Sudan) [21]. Moreover, two thirds of participants in the Logo health zone met the OAE criteria. A positive correlation between the frequency of seizures and MF density supports recent findings from a cohort study in Cameroon which showed that the risk to develop epilepsy increases with increasing intensity of childhood infection with *O*. *volvulus* [5]. In that cohort study, the population attributable fraction of epilepsy associated with onchocerciasis was estimated at 91.7% [5], and PWE in the investigated villages had similar clinical manifestations as observed in our study [22].

By meticulously taking the history of our study participants, we were able to identify 32 PWE who reported experiencing nodding seizures. They all met the criteria of the consensual case definition of probable nodding syndrome [23]. PWE who experienced nodding seizures in our study were younger, more often cognitively impaired and had more food-triggered seizures; all these clinical aspects align with the nodding syndrome definition [23]. In addition, the description of the 12 PWE with Nakalanga features presented in Table 3 closely matched previous reports from other African countries [18]. PWE with Nakalanga features were more often of short stature, cognitively impaired, onchocerciasis-infected and with very frequent seizures. Therefore, both nodding and Nakalanga syndromes appear to be the severe forms of OAE. The fact that these phenotypic presentations have only been reported in onchocerciasis-endemic settings until now strongly suggests the role of *O*. *volvulus* in triggering these conditions.

In the multivariable model, high MF density was associated with more frequent seizures in Aketi only. The fact that only onchocerciasis-infected PWE were recruited in Aketi cannot explain these results, because the analysis of skin snip-positive participants in Logo did not reveal an association between seizures and MF (see S2 File). We however noticed the significant seizure-reducing effect of AED in Logo compared to Aketi (Table 5). Although similar proportions of participants had previously used AED in both study sites, previous surveys by our team showed that in Logo, 22.6% of PWE took AED regularly [9] compared to only 9.2% in Aketi [10]. Therefore, it is conceivable that the better AED adherence in the Logo health zone could mask an association between MF and seizures. Another observation emerging from the multivariable analysis is the inverse relationship between seizures and age of PWE in the Logo health zone, suggesting that OAE is more severe among younger PWE.

Stunting was a frequent trait among PWE in the Logo health zone, irrespective of skin snip status. Although growth retardation is a common feature in persons with OAE including nodding syndrome [7,22,24], other factors such as undernutrition and poverty observed among PWE may contribute to this condition as reported in an Ethiopian study [25]. However, given that we did not investigate the feeding habits of our participants, our study is unable to confirm this.

Participants with a family history of epilepsy had a higher prevalence and intensity of *O*. *volvulus* infection. This suggests a greater exposure to onchocerciasis and explains the clustering of PWE in such households, which is a characteristic feature of OAE [7]. This is in line with previous reports of villages and families who are closer to blackfly breeding sites having more PWE [3,11,19,21]. Two studies conducted in the DRC also reported a high frequency of family history of epilepsy [24,26]. One of these studies was performed in onchocerciasis-endemic villages in the Bas-Congo province, while the other was done in a reference epilepsy treatment centre in Lubumbashi which probably served some PWE from surrounding endemic villages. Although the latter study mentioned a possible genetic cause [26], onchocerciasis is a more likely explanation for the family clustering of PWE that was observed.

While this was not the purpose of the study, we noted some discrepancies in the onchocerciasis diagnosis using skin snips (reference technique in our study) and Ov16 rapid tests (Table 2); the rapid tests yielded 23.2% of false positives. Rapid tests may therefore not be optimal for diagnosing ongoing *O*. *volvulus* infection, but they provide information about exposure to the parasite. These tests remain key and convenient for field use when assessing onchocerciasis transmission by testing children aged 10 years and below, as was the case in Cameroon [19], Nigeria [27], DRC [10] and Tanzania [20].

### Limitations of the study

Our study has several limitations. Laboratory and imaging investigations to exclude other possible causes of epilepsy such as neurocysticercosis were not performed. However, previous studies had suggested that *Taenia solium* infection is not prevalent in the Logo Health zone [4] nor in the Bas-Uélé province [28]. In addition, the high proportion of PWE meeting the OAE criteria makes it unlikely for another infectious pathology to be the main reason behind the high epilepsy prevalence. Another limitation is the fact that seizure information and past history of participants were obtained by interviewing family members, and could be subject to recall bias. Absence seizures and some focal seizures which are more subtle may have been under-reported as a consequence. Moreover, cognitive function was not assessed using a validated series of tests.

In conclusion, PWE in onchocerciasis-endemic villages in the Logo Health zone presented with wide clinical spectrum including generalized seizures, nodding seizures, Nakalanga features and other OAE characteristics. MF density was significantly and positively associated with seizure frequency in Aketi. It is expedient that onchocerciasis control measures be strengthened to prevent new OAE cases, while providing comprehensive care to confirmed PWE using appropriate AED and cognitive rehabilitation services. The possible added value of anti-filarial drugs in the treatment of OAE including nodding syndrome is currently being investigated [12,29].

## Supporting information

S1 File STROBE checklist. (PDF)

S2 File Multivariable analysis of skin snip-positive participants from Logo. (PDF)
