# Supplementary material for: Onchocerciasis-associated epilepsy in the Democratic Republic of Congo: Clinical description and relationship with microfilarial density
Source: PLoS Negl Trop Dis. 2019 Jul 17;13(7):e0007300. doi: 10.1371/journal.pntd.0007300 (PMC6663032; doi:10.1371/journal.pntd.0007300)
Supplement: S1 File — (PDF) [file pntd.0007300.s001.pdf]

S1 File. STROBE checklist for the study: "Onchocerciasis-associated epilepsy in the Democratic Republic of Congo: Clinical description and relationship with microfilarial density" (Siewe et al.)

	Item No.	Recommendation	Location in manuscript	Relevant text from manuscript
**Title and abstract**	1	(*a*) Indicate the study's design with a commonly used term in the title or the abstract	Title page	"Clinical description of onchocerciasis-associated epilepsy" in the title
		(*b*) Provide in the abstract an informative and balanced summary of what was done and what was found	Abstract, paragraphs 2-3	Positive correlations between seizure frequency and microfilarial density were observed
**Introduction**				
Background/rationale	2	Explain the scientific background and rationale for the investigation being reported	Introduction, paragraphs 1-2	Recent studies in the DRC have revealed a high epilepsy prevalence in hyper-endemic onchocerciasis foci
Objectives	3	State specific objectives, including any prespecified hypotheses	Introduction, paragraph 2	In a bid to further elucidate the association between epilepsy and onchocerciasis…
**Methods**				
Study design	4	Present key elements of study design early in the paper	Methods, paragraph 1	We carried out a cross-sectional, descriptive study…
Setting	5	Describe the setting, locations, and relevant dates, including periods of recruitment, exposure, follow-up, and data collection	Methods, paragraph 2	The study was carried out in two health zones in the DRC, namely Logo (in the Ituri province) and Aketi (in the Bas-Uélé province).
Participants	6	(*a*) *Cohort study*—Give the eligibility criteria, and the sources and methods of selection of participants. Describe methods of follow-up<br>*Case-control study*—Give the eligibility criteria, and the sources and methods of case ascertainment and control selection. Give the rationale for the choice of cases and controls<br>*Cross-sectional study*—Give the eligibility criteria, and the sources and methods of selection of participants	Methods, paragraph 8	Epilepsy diagnosis was confirmed according to the 2014 International League Against Epilepsy (ILAE) operational definition: two or more unprovoked seizures with at least 24 hours separating the two events.
		(*b*) *Cohort study*—For matched studies, give matching criteria and number of exposed and unexposed<br>*Case-control study*—For matched studies, give matching criteria and the number of controls per case		NA

Variables	7	Clearly define all outcomes, exposures, predictors, potential confounders, and effect modifiers. Give diagnostic criteria, if applicable	Methods, paragraphs 8-9	The main outcome variables were microfilarial density, seizure frequency and seizure-related morbidities. Onchocerciasis diagnosis was by skin snip, and epilepsy diagnosis according to the ILAE 2014 definition.
Data sources/ measurement	8	For each variable of interest, give sources of data and details of methods of assessment (measurement). Describe comparability of assessment methods if there is more than one group	Methods, paragraphs 8-9	- The evaluation of the seizure frequency included all diagnosed seizure types.<br>- Skin snips were taken from the left and right iliac crests… Mf densities were expressed as mf/skin snip
Bias	9	Describe any efforts to address potential sources of bias	Limitations, paragraph 1	… seizure information and past history of participants were obtained by questioning the PWE and caretakers, and could be subject to recall bias
Study size	10	Explain how the study size was arrived at	Methods, paragraphs 3-4	All persons suspected to have epilepsy who reported to the mobile clinics were briefed… Upon confirmation of the epilepsy diagnosis, PWE were further interviewed and examined.

Quantitative variables	11	Explain how quantitative variables were handled in the analyses. If applicable, describe which groupings were chosen and why	Methods, paragraph 10	Sub-section: Data analysis
Statistical methods	12	(*a*) Describe all statistical methods, including those used to control for confounding	Methods, paragraph 10	Sub-section: Data analysis
		(*b*) Describe any methods used to examine subgroups and interactions	Methods, paragraph 10	Sub-section: Data analysis
		(*c*) Explain how missing data were addressed	Results, paragraph 3	The denominators may vary for the different parameters because PWE with missing data were excluded
		(*d*) *Cohort study*—If applicable, explain how loss to follow-up was addressed<br>*Case-control study*—If applicable, explain how matching of cases and controls was addressed<br>*Cross-sectional study*—If applicable, describe analytical methods taking account of sampling strategy	NA	NA
		(*e*) Describe any sensitivity analyses	NA	NA

**Results**				
Participants	13*	(a) Report numbers of individuals at each stage of study—eg numbers potentially eligible, examined for eligibility, confirmed eligible, included in the study, completing follow-up, and analysed	Results, paragraphs 1-7	- A total of 420 PWE in the Logo health zone were enrolled<br>- All 81 PWE recruited in the Aketi health zone…
		(b) Give reasons for non-participation at each stage	Methods, paragraph 7	… seizure frequencies were obtained from participants with positive skin snips
		(c) Consider use of a flow diagram	NA	NA
Descriptive data	14*	(a) Give characteristics of study participants (eg demographic, clinical, social) and information on exposures and potential confounders	Results, paragraphs 1,11	Tables 1 and 2
		(b) Indicate number of participants with missing data for each variable of interest	Results, paragraphs 2, 3	Tables 2 and 4
		(c) *Cohort study*—Summarise follow-up time (eg, average and total amount)	NA	NA
Outcome data	15*	*Cohort study*—Report numbers of outcome events or summary measures over time	NA	NA
		Case-control study—Report numbers in each exposure category, or summary measures of exposure	NA	NA
		Cross-sectional study—Report numbers of outcome events or summary measures	Results, paragraphs 1-7	Sub-sections: "PWE in the Logo Health Zone" and "PWE in the Aketi Health Zone"
Main results	16	(*a*) Give unadjusted estimates and, if applicable, confounder-adjusted estimates and their precision (eg, 95% confidence interval). Make clear which confounders were adjusted for and why they were included	Results, paragraphs 1-7	Tables 1, 2, 4, 5
		(*b*) Report category boundaries when continuous variables were categorized	NA	NA
		(*c*) If relevant, consider translating estimates of relative risk into absolute risk for a meaningful time period	NA	NA
Other analyses	17	Report other analyses done—eg analyses of subgroups and interactions, and sensitivity analyses	NA	NA
**Discussion**				

Key results	18	Summarise key results with reference to study objectives	Discussion, paragraph 1	A wide spectrum of seizures was observed… A positive correlation between the frequency of seizures and mf density supports recent findings from a cohort study…
Limitations	19	Discuss limitations of the study, taking into account sources of potential bias or imprecision. Discuss both direction and magnitude of any potential bias	Limitations, paragraph 1	Our study has several limitations…
Interpretation	20	Give a cautious overall interpretation of results considering objectives, limitations, multiplicity of analyses, results from similar studies, and other relevant evidence	Discussion, paragraph 1	A positive correlation between the frequency of seizures and mf density supports recent findings from a cohort study in Cameroon which showed that the risk to develop epilepsy increases with increasing intensity of childhood infection with *O. volvulus*
Generalisability	21	Discuss the generalisability (external validity) of the study results	Discussion, paragraphs 1-9	Nodding seizures and Nakalanga features were reported, suggesting a high prevalence of OAE in these communities as previously observed in Ituri (DRC), in the Mbam valley (Cameroon), Mahenge (Tanzania), and Maridi (South Sudan)
**Other information**				
Funding	22	Give the source of funding and the role of the funders for the present study and, if applicable, for the original study on which the present article is based		This research was funded by the European Research Council, Grant number 768815
