# Supplementary material for: Onchocerciasis-associated epilepsy in the Democratic Republic of Congo: Clinical description and relationship with microfilarial density
Source: PLoS Negl Trop Dis. 2019 Jul 17;13(7):e0007300. doi: 10.1371/journal.pntd.0007300 (PMC6663032; doi:10.1371/journal.pntd.0007300)
Supplement: S2 File — (PDF) [file pntd.0007300.s002.pdf]

Supplementary Material S2

Multivariate analysis of skin snip positive PWE in Logo (n=143)

	**Skin snip positive PWE (Logo)**	
	Adj. IRR (95% CI)	*P-value*
MF density	1.002 (0.999–1.005)	*0.205*
Age	0.995 (0.977–1.016)	*0.633*
Female gender	0.466 (0.284–0.765)	***0.001***
Previous AED treatment	0.648 (0.371–1.114)	*0.091*

MF: Microfilariae
AED: Anti-epileptic drug
Adj. IRR: Adjusted incidence risk ratio
CI: Confidence interval
